# Seasonal dynamics of marine snow‐associated and free‐living demethylating bacterial communities in the coastal northern Adriatic Sea

**DOI:** 10.1111/1758-2229.12783

**Published:** 2019-07-25

**Authors:** Paul A. Steiner, Eva Sintes, Rafel Simó, Daniele De Corte, Daniela Marić Pfannkuchen, Ingrid Ivančić, Mirjana Najdek, Gerhard J. Herndl

**Affiliations:** ^1^ Limnology and Bio‐Oceanography, Center of Functional Ecology University of Vienna, Althanstrasse 14 1090 Vienna Austria; ^2^ Institut de Ciències del Mar, ICM‐CSIC, Pg Marítim de la Barceloneta 37‐49 08003 Barcelona Catalonia Spain; ^3^ Department of Subsurface Geobiological Analysis and Research Japan Agency for Marine‐Earth Science and Technology, Natushima 2‐15 Yokosuka Kanagawa Japan; ^4^ Center for Marine Research Ruder Boskovic Institute, G. Paliaga 5 52210 Rovinj Croatia; ^5^ NIOZ, Department of Marine Microbiology and Biogeochemistry Royal Netherlands Institute for Sea Research, Utrecht University, PO Box 59, Alberta Den Burg 1790 The Netherlands

## Abstract

The extent of DMSP demethylation has been hypothesized to depend on DMSP availability and bacterial sulfur demand, which might lead to niche differentiation of the demethylating bacterial community. In this study, we determined DMSP concentrations in marine snow and the ambient water over a seasonal cycle and linked DMSP concentrations to the abundance of bacteria harbouring the demethylation *dmd*A gene in the Adriatic Sea. In marine snow, DMSP concentrations were up to four times higher than in the ambient water and three times higher in marine snow in summer than in winter. The average *dmd*A:*rec*A gene ratio over the sampling period was 0.40 ± 0.24 in marine snow and 0.48 ± 0.21 in the ambient water. However, at the subclade level, differences in the demethylating bacterial community of marine snow and the ambient water were apparent. Seasonal patterns of potentially demethylating bacteria were best visible at the oligotype level. In the ambient water, the SAR116 and the OM60/NOR5 clade were composed of oligotypes that correlated to high DMSP concentrations, while oligotypes of the *Rhodospirillales* correlated to low DMSP concentrations. Our results revealed a pronounced seasonal variability and spatial heterogeneity in DMSP concentrations and the associated demethylating bacterial community.

## Introduction

Dimethylsulfoniopropionate (DMSP) is a sulphur‐containing metabolite mainly produced by marine phytoplankton in species‐specific concentrations (Stefels, 2000). DMSP acts primarily as an osmolyte but also serves as an antioxidant, cryoprotectant or represents an overflow metabolite (Karsten *et al*., 1996; Malin and Erst, 1997; Stefels, 2000; Simó, 2001; Sunda *et al*., 2002). It is released from phytoplankton into the ambient water and is subsequently degraded by bacteria either via the DMSP cleavage or demethylation pathway (Kiene *et al*., 2000; Moran *et al*., 2012). Diverse taxa of marine bacteria can mediate one or the other, or both processes (Simó, 2001; Reisch *et al*., 2011; Moran *et al*., 2012; Varaljay *et al*., 2015; Nowinski *et al*., 2019), thereby controlling the fate of dissolved DMSP in the ocean. Genes of the demethylation pathway have been found in 2‐ to about 5‐fold higher abundance than genes of the cleavage pathway in metagenomes obtained from the Global Ocean Survey (Moran *et al*., 2012), at Station ALOHA (Varaljay *et al*., 2012) and Monterey Bay (Nowinski *et al*., 2019). Approximately 90% of dissolved DMSP in the ocean is demethylated (Kiene *et al*., 2000; Kiene and Linn, 2000a), implicating that the sulfur moieties are not lost to the atmosphere via dimethylsulfide (DMS), but rather enter the marine food web (Moran *et al*., 2012). Total DMSP concentrations in the ocean depend largely on the abundance and intracellular DMSP concentrations of the main DMSP‐producing phytoplankton taxa (Keller *et al*., 1989) but also on environmental factors such as salinity, grazing pressure and nutrient concentrations (Stefels *et al*., 2007). Responses of demethylating bacteria to phytoplankton blooms (Howard *et al*., 2011), specific phytoplankton taxa (Howard *et al*., 2011; Varaljay *et al*., 2015) and to DMSP concentrations (Frade *et al*., 2016; Nowinski *et al*., 2019) indicate that demethylating bacteria can occupy a wide range of niches. Specific subclades of the *dmd*A gene, responsible for the demethylation of DMSP, have been found in different size fractions of coastal waters, suggesting that bacteria with contrasting lifestyles, such as free‐living *versus* particle‐attached, could potentially demethylate DMSP (Varaljay *et al*., 2010).

In this study, we analysed the spatial and temporal distribution patterns of the demethylating bacterial community and determined DMSP concentrations in marine snow (MS) and the ambient water (AW) over a seasonal cycle. We expected substantially higher DMSP concentrations in MS than in the AW as MS originates mainly from transparent exopolymeric particles (TEPs) released by phytoplankton into the ambient water where they randomly collide (Herndl, 1992; Alldredge *et al*., 1993; Schuster and Herndl, 1995). To determine the factors affecting DMSP concentrations in MS and the AW, we characterized the phytoplankton community and measured contextual environmental parameters over a seasonal cycle. We hypothesized that the demethylating bacterial community in MS differs from that in the AW and varies over seasons. Changes in the bacterial community might have important implications for the cycling of DMSP, as demethylation of DMSP prevents the formation of the climatically relevant gas DMS (Moran *et al*., 2012).

## Results

### *Phytoplankton and DMSPt + DMS concentrations*

Four main phytoplankton groups (diatoms, silicoflagellates, dinoflagellates and coccolithophores) were present during the seasonal cycle (Supporting Information Fig. S1). Total phytoplankton abundance integrated over the upper 10 m of the ambient water (AW) was highest on 15 November (1.1 × 10^9^ cells m^−2^) due to a diatom bloom (8.7 × 10^8^ cells m^−2^) and high abundance of coccolithophores (1.6 × 10^8^ cells m^−2^) (Supporting Information Fig. S1). Diatoms were generally the most abundant phytoplankton group. Coccolithophores were abundant on 15 June (1.1 × 10^8^ cells m^−2^) and 19 July (1.4 × 10^8^ cells m^−2^). Silicoflagellates were the least abundant group (Supporting Information Fig. S1).

**Figure 1:**
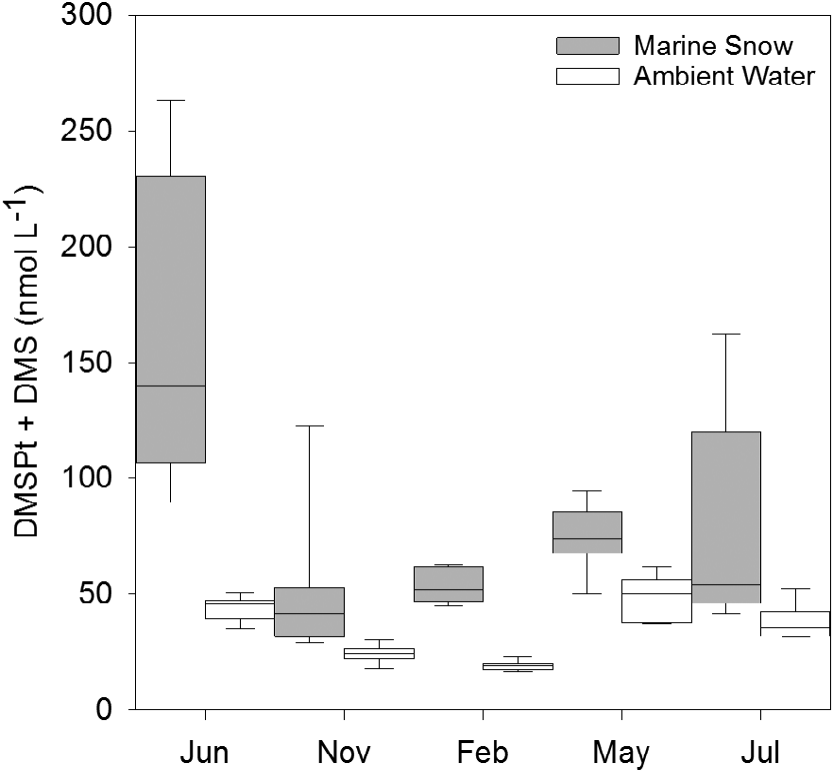
Box‐plot of DMSP total + DMS concentrations (median, 10th, 25th, 75th and 90th percentiles) in marine snow (grey) as compared to the ambient water (white).

Particulate and dissolved DMSP + DMS (DMSPt + DMS) concentrations averaged over all samples were significantly higher in marine snow (MS) (80.46 ± 52.44 nmol L^−1^) than in AW (33.62 ± 12.57 nmol L^−1^) with the monthly averaged enrichment factor (EF) ranging from 2 to 4 (Fig. 1). DMSP concentrations in MS and the AW showed seasonal variations with lowest monthly average in November and February and highest concentrations in May, June and July (Fig. 1 and Supporting Information Table S1).

### *dmdA subclades determined by qPCR*

**Figure 2:**
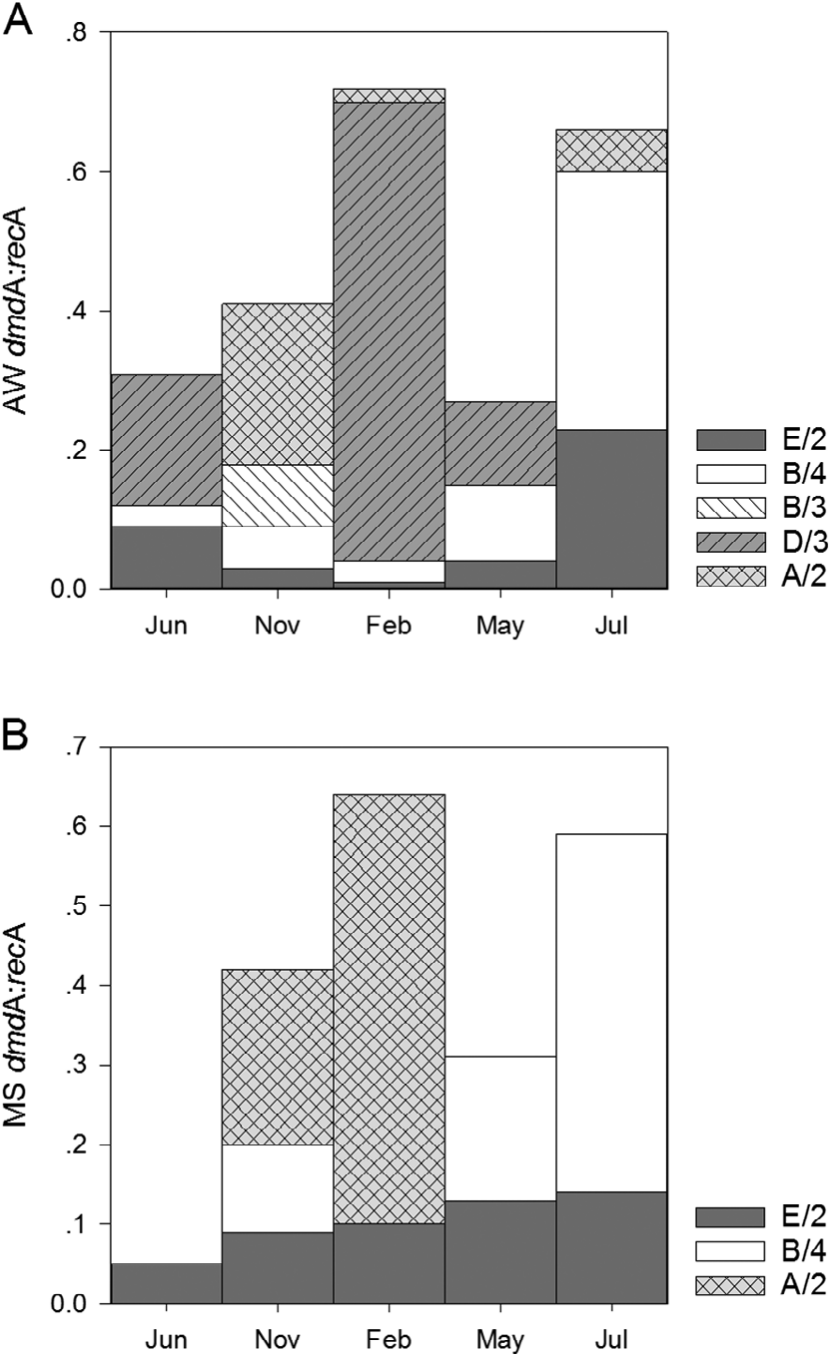
Seasonal variation of the monthly average of *dmd*A subclades (E/2, B/4, B/3, D/3, and A/2) normalized to *rec*A gene abundance assessed by qPCR in AW (A) and MS (B). SD was always below 27% of the respective subclades normalized to recA gene abundance.

Five of the nine specific primer pairs (A/2, B/3, B/4, D/3 and E/2) for the *dmd*A subclades generated clear signals in PCR and hence, were subsequently used for qPCR. On average, 40.0 ± 24% of the bacterial community in MS and 48 ± 21% of the AW bacterial community harboured the *dmd*A gene. Subclades D/3 and B/3 were only detected in the AW. The *dmd*A:*rec*A ratio of subclade D/3 was on average 0.19 ± 0.27, while subclade B/3 had the lowest (0.02 ± 0.04) contribution to the total demethylating bacterial community (Fig. 2). The subclades E/2 and B/4 contributed similarly to the DMSP demethylating bacterial community in AW and MS (Fig. 2). Subclade E/2 was significantly more abundant in June than in any other month. The relative abundance of subclade A/2 was approximately twice as high in MS (0.15 ± 0.24) than in AW (0.06 ± 0.1). Subclade A/2 was mainly present in November and February in MS and AW (Fig. 2).

### *Bacterial community composition*

**Figure 3:**
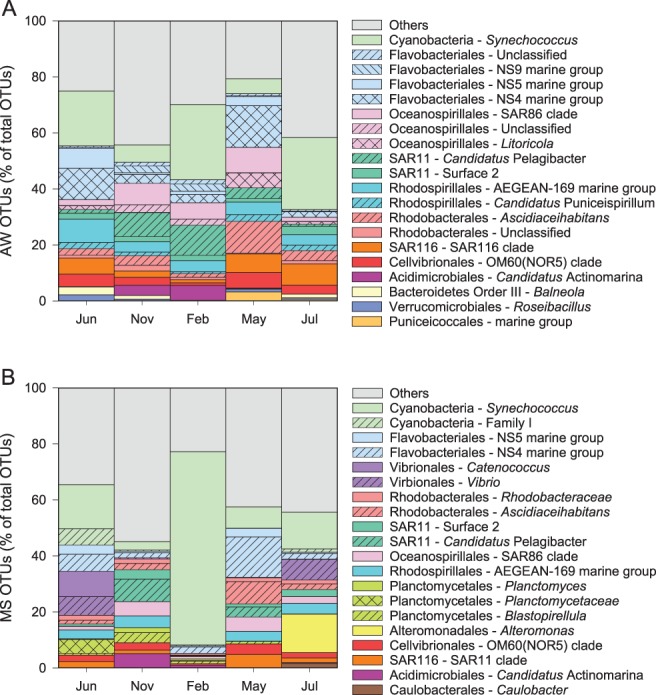
Relative contribution of the 20 most abundant OTUs in the AW (A) and MS (B) expressed as the percentage of total OTUs averaged over each month. All other OTUs are summed up and plotted together as ‘Others’ in grey.

All bacterial groups specifically targeted by the qPCR primers of the *dmd*A subclades A/2, B/3, B/4, D/3 and E/2 were observed in both, the AW and MS. Clade A primers target *Rhodospirillales* and *Roseobacter*. *Roseobacter* was present only in MS and accounting for 0.1% of the OTUs there, hence subclade A/2 was mostly represented by *Rhodospirillales*. The SAR11 clade (with members representative for *dmd*A subclade D/3) amounted in total to 4.6% in MS and 10.6% in AW, *Rhodospirillales* (with members representing the subclade A/2) contributed 3.5% to MS and 6.2% to AW, the SAR116 clade (with members representative for subclades B/3 and B/4) constituted 3.4% to MS and 5.7% to AW, and the OM60 clade (with members representative for subclade E/2) contributed 2.5% in MS and 3.3% in AW (Fig. 3).

**Figure 4:**
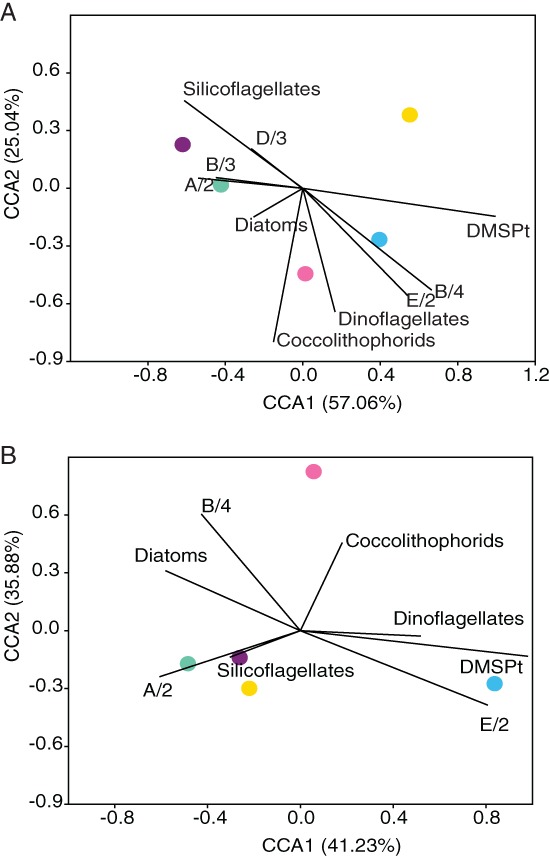
Canonical correspondence analysis (CCA) based on the seasonal variation of the bacterial community in the AW (A) and MS (B). The percentage of variation of the bacterial community explained by each axis is shown in parenthesis in the axis title. *Synechococcus* was excluded from the CCAs. Environmental variables (DMSPt concentration, diatoms, coccolithophores, dinoflagellates, silicoflagellates abundances and the *dmd*A subclades E/2, B/4, B/3, D/3 and A/2 relative gene abundances) are depicted as triplot. The colour‐coding for the sampling months is as follows: cyan: June; green: November; purple: February; yellow: May; pink: July.

The variation of the bacterial community composition in MS and the AW in June and July coincided with the abundance of dinoflagellates, coccolithophores and DMSPt + DMS concentrations and the abundance of the *dmd*A subclade E/2 (Fig. 4A and B). In contrast, silicoflagellates, diatoms and the subclades B/3, D/3 and A/2 corresponded to the AW community composition in November and February (Fig. 4A). Between November and May, the bacterial community in MS was related to a higher contribution of diatoms, silicoflagellates and the *dmd*A subclade A/2. The MS bacterial community in July corresponded mainly to the *dmd*A subclade B/4 (Fig. 4B).

**Figure 5:**
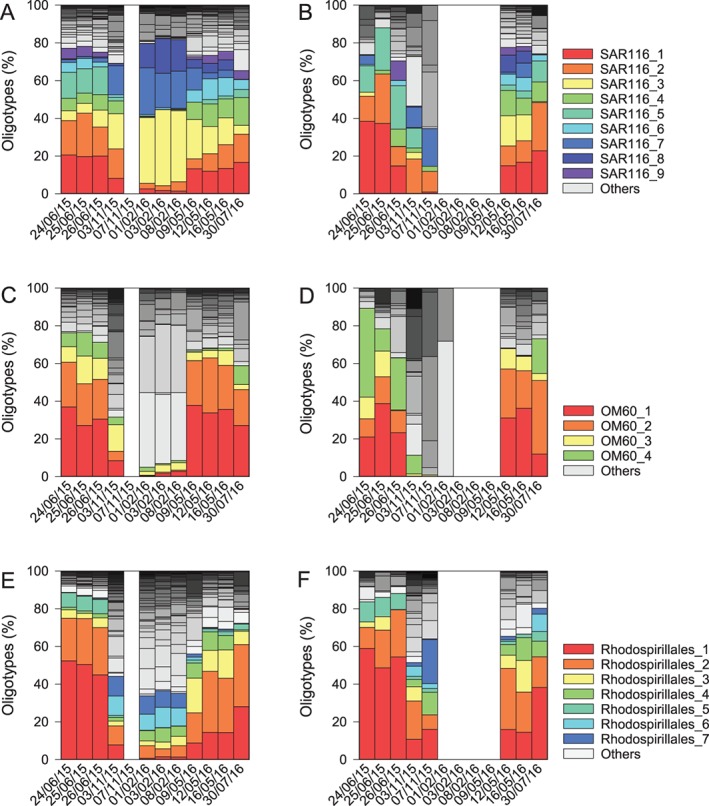
Distribution of oligotypes expressed as the percentage of the corresponding group for each sampling day: SAR116 oligotypes in AW (A); SAR116 oligotypes in MS (B); OM60 oligotypes in AW (C); OM60 oligotypes in MS (D); Rhodospirillales oligotypes in AW (E); Rhodospirillales oligotypes in MS (F). All oligotypes with a total abundance larger than 1000 reads over all sampling days are displayed in colour and numbered, oligotypes with less than a 1000 reads are depicted in grey and summed up as ‘Others’.

The OTUs targeted by the primers of the *dmd*A subclades and appearing in both, MS and AW, were further analysed with oligotyping to obtain more detailed insights into their temporal distribution and preferred life styles. Oligotyping revealed 40 SAR116 oligotypes, 26 OM60 clade oligotypes and 48 Rhodospirillales oligotypes (Fig. 5). In the AW, the 9 most abundant oligotypes of the SAR116 clade (representative of subclade B/4) exhibited seasonal dynamics. Oligotypes SAR116_3, 7 and 8 were more abundant in November and February and oligotypes SAR116_1, 2, 4, 5, 6 and 9 were more abundant in June, July and May (Fig. 5A). Oligotype SAR116_6 and SAR116_9 correlated with DMSPt + DMS concentrations in the AW (Supporting Information Fig. S2). The dynamics of the MS oligotypes of SAR116 showed a similar seasonal pattern as in the AW, however, in a less pronounced way (Fig. 5B). Oligotype SAR116_5 correlated significantly to DMSPt + DMS concentrations in MS (Supporting Information Fig. S2). The Gammaproteobacteria clade OM60, representative of demethylating subclade E/2 bacteria, was present in all seasons with highest relative abundances in May (Fig. 3). The oligotypes OM60_1 and OM60_2 correlated significantly with DMSPt + DMS concentrations. OM60 oligotypes did not correlate with DMSPt + DMS concentrations in MS (Supporting Information Fig. S2). The four most abundant oligotypes were specifically dominant in May, June and July (Fig. 5C and D). *Rhodospirillales* and *Roseobacteriales* are the representative group of subclade A/2; however, *Roseobacter* was not present in the AW. The most abundant OTU was AEGEAN‐169 in the AW in February (Fig. 3A). The *Rhodospirillales* oligotypes showed a strong seasonality in the AW with oligotypes Rhodospirillales_6 and Rhodospirillales _7 appearing in November and February, while all others were more abundant in June, May and July (Fig. 5E). Oligotypes Rhodospirillales_6 and Rhodospirillales_7 were negatively related to DMSPt + DMS concentrations (Supporting Information Fig. S2). The seasonality of this group was also less pronounced in MS than in AW (Fig. 5F). Oligotypes Rhodospirillales_1 and Rhodospirillales_5 correlated significantly to DMSPt + DMS concentrations in MS (Supporting Information Fig. S2).

## Discussion

### *Demethylating bacterial community in ambient water versus marine snow*

Typically, approximately 90% of DMSP occurs in plankton cells, and only between 2.3% and 10% is actually dissolved (Kiene and Slezak, 2006). The DMS fraction of the measured DMSPt + DMS is presumably small as average DMS concentrations of 12 nmol L^−1^ in a coccolithophore bloom in the Northeast Atlantic represent rather high concentrations (Malin *et al*., 1993). The DMSPt + DMS concentrations in AW were comparable to previously reported concentrations for the coastal Mediterranean Sea (Belviso *et al*., 1990; Vila‐Costa *et al*., 2008). DMSPt + DMS in MS reached high concentrations, comparable to concentrations measured in phytoplankton blooms (Matrai and Keller, 1993). The observed high DMSPt + DMS concentrations in MS (Fig. 1) are likely due to DMSP accumulation caused by high abundance of MS‐associated phytoplankton (Turner, 2002). Higher DMSP concentrations in MS, however, might also be caused by lower DMSP degradation rates. The overall bacterial activity determined as leucine incorporation was up to 7 times higher in MS as compared to the AW (Supporting Information Fig. S2B and Table S1), indicating a highly active bacterial community in MS. Consequently, it is unlikely that lower DMSP degradation rates are not responsible for the higher DMSP concentrations in MS. Bacterial activity has been shown to correlate with dissolved DMSP turnover rate (Kiene and Linn, 2000b). Hence, despite potentially high dissolved DMSP turnover, DMSP accumulated in MS. However, further work is required to determine dissolved DMSP concentrations and turnover rates to obtain better insights into the dynamics of DMSP utilization.

While the contribution of demethylating bacteria to the bulk bacterial community was similar in MS and AW (40% and 48%, respectively) and over the seasons, with only few exceptions, the composition of the demethylating bacterial community varied considerably between MS and AW (Fig. 2A and B). The *dmd*A subclades D/3 and B/3 exclusively occurred in the AW, suggesting that these subclades of demethylating bacteria prefer a free‐living lifestyle (Fig. 2A and B). This is in agreement with the known representative of subclade D/3, the SAR11 clade (Acinas *et al*., 1999). However, it contradicts the typically particle‐associated lifestyle of the SAR116 clade (Salazar *et al*., 2015; Jain and Krishnan, 2017) affiliated to subclades B/3 and B/4. Subclade B/3 was significantly more abundant in the free‐living fraction in coastal waters of the south eastern United States, indicating that some members of the SAR116 clade, particularly the OTUs capable of demethylation, might exhibit a free‐living life style (Varaljay *et al*., 2010). The *dmd*A subclade A/2 was twice as abundant in MS than in AW (Table S1). This is in agreement with the finding of the demethylating subclade A/2 colonizing the surface mucus of corals (Frade *et al*., 2016) but contradicts the findings of Varaljay *et al*. (2010) indicating that subclade A/2 is composed of bacteria with contrasting lifestyles. The high abundance of the *dmd*A subclade A/2 and the low abundance of *Rhodospirillales* in MS further indicate that this subclade is composed of diverse taxa (Figs. 2B and 3B). In this study, the most abundant *Rhodospirillales* OTU was affiliated to the AEGEAN‐169 marine group (Fig. 3A), typically found in the AW (Salazar *et al*., 2015). Subclades E/2 and B/4 occurred in both, MS and AW in this coastal system, similarly as previously reported for the south eastern U.S. coast (Varaljay *et al*., 2010). Taken together, the *dmd*A gene‐harbouring bacterial community constitutes a similar fraction to the total bacterial community in MS as in AW. However, the community composition of the *dmd*A gene harbouring community on the subclade level differs substantially between MS and AW. AW and MS are characterized by contrasting DMSPt + DMS concentrations, which might favour niche differentiation of demethylating bacteria. However, the observed differences in MS‐associated and free‐living demethylating communities also reflect phylogenetically conserved general patterns of particle‐attached and free‐living bacteria (Salazar *et al*., 2015).

### *Temporal variability of the demethylating bacterial community*

Seasonal dynamics of the demethylating bacterial community were evident on the *dmd*A subclade level for most subclades (Fig. 2A and B). Some subclades appeared episodically throughout the year indicating that the fluctuations in the targeted bacterial groups are not necessarily related to environmental changes on a seasonal scale but related to sporadic events. The northern Adriatic Sea is a highly dynamic system, as indicated by the variation in the nutrient concentrations over the investigation period (Supporting Information Fig. S3) and as shown previously (Cantoni *et al*., 2003; Cozzi *et al*., 2004). The appearance of subclade B/3 might not be related to seasonal changes but rather to a diatom bloom in November (Supporting Information Fig. S1). The patchy distribution of subclade D/3 in the AW might also indicate that the representative bacterial group (SAR11 clade) consists of many ecotypes with fine‐tuned adaptations to physical and chemical gradients (Carlson *et al*., 2009). The SAR11 clade consists of members highly active in DMSP degradation correlating with haptophyte abundance (including coccolithophores) (Nowinski *et al*., 2019). In this study, subclade D/3 was present in June, when coccolithophores were abundant and DMSPt + DMS concentrations high. However, subclade D/3 was also present in February, coinciding with high relative abundance of SAR11 16SrRNA genes (Fig. 3A). Seasonality was observed in the temporal distribution of the *dmd*A subclades E/2 and B/4 in the AW and subclade A/2 present mostly in November and February in MS and AW (Fig. 2). The gammaproteobacterial clade OM60, targeted by E/2 primers, has been found in nutrient rich coastal areas (Xia *et al*., 2015) and was shown to vary with DMSPt + DMS concentrations (Nowinski *et al*., 2019). In this study, the relative abundance of subclade E/2 correlated to DMSPt + DMS concentrations in MS (*r* = 0.89, *p* < .05) (Fig. 4B). Subclade A/2 occurred mostly in samples with low DMSPt + DMS concentrations in MS and AW pointing to diverse lifestyles within this subclade as reported in other studies (Varaljay *et al*., 2010; Frade *et al*., 2016; Nowinski *et al*., 2019). Conclusions based on normalized bacterial communities might not reflect actual community dynamics. In this study, however, the bacterial abundance remained fairly stable over the seasons in the AW. MS exhibited significantly higher prokaryotic abundance only in November as compared to all other months (Supporting Information Fig. S4). The rather stable bacterial abundance over seasons strengthens the conclusions drawn from normalized gene abundances.

The phenomenon of seasonal variation of closely related bacteria has been observed previously in other studies (Yung *et al*., 2015; Ward *et al*., 2017). However, here we show for the first time seasonality of potentially demethylating bacteria at the oligotype level. We documented a seasonal switch of potentially demethylating oligotypes along with changes in environmental factors such as DMSPt + DMS concentrations (Figs. 1 and 5 and Supporting Information Table S1). The high abundance of potentially demethylating oligotypes and the significant correlations to DMSPt + DMS concentrations of some oligotypes (Supporting Information Fig. S2) suggest a wide range of niches for DMSP demethylation. DMSP demethylation of oligotypes at various DMSP concentrations might have major biogeochemical and ecological implications, by preventing the formation of the climatically relevant gas DMS and by retaining the sulphur compounds in the marine food web (Moran *et al*., 2012). While closely related *dmd*A gene harbouring oligotypes revealed a strong seasonality in their occurrence, their preferential lifestyle (free‐living vs. particle‐associated) was less obvious using oligotyping. This stresses the finding of the particle‐association lifestyle as a deeply rooted trait in the phylogenetic tree (Salazar *et al*., 2015) and suggests that traits defining a narrow niche might have only recently been acquired. A less obvious differentiation of lifestyles between oligotypes might have also been caused by the method used to collect MS with syringes, resulting in a dilution of MS with small amounts of AW including the pore water of MS (supplementary video). This inefficient separation of MS and AW might have caused a less pronounced differentiation of free‐living versus particle‐associated oligotypes. However, a strict separation of MS and AW is difficult unless the pore water (including biological and chemical compounds) is removed by filtration. Moreover, the bacterial community is not strictly separated into free‐living and particle‐associated bacteria, but rather into a gradient of particle association and sporadic attachment and detachment (Gibiansky *et al*., 2010; Salazar *et al*., 2015; Son *et al*., 2015).

The existence of two competing DMSP degradation pathways leading to contrasting ecologically and climatically relevant compounds has raised the question on the mechanisms controlling the ‘bacterial switch’ to one or the other pathway (Cantoni and Anderson, 1956; Kiene *et al*., 2000; Moran *et al*., 2012). Sulphur demand and DMSP availability have been suggested to control DMSP demethylation, while sulphur saturation and carbon demand have been suggested to control the cleavage pathway (Kiene *et al*., 2000; Simó, 2001). UV radiation has also been hypothesized to affect the ‘bacterial switch’ and the fate of sulphur (Vallina and Simó, 2007; Levine *et al*., 2012) as DMS acts as a radical scavenger (Brugger *et al*., 1998; Sunda *et al*., 2002; Levine *et al*., 2012). However, it was also suggested that even at high DMSP concentrations demethylating and DMSP‐cleaving bacteria compete (Levine *et al*., 2012), contrasting the scenario formulated in the sulphur‐demand hypothesis (Kiene *et al*., 2000). In this study, we analysed DMSP demethylating bacteria and environmental factors potentially shaping the demethylating community. Our results suggest that MS might be a hotspot of competition between demethylating and DMSP cleaving bacteria due to high DMSPt + DMS concentrations and high abundance of demethylating bacteria. However, further work is needed to determine the potential for DMSP cleavage and the abundance of demethylation and cleavage gene transcripts to test this assumption. We confirmed the seasonality of the demethylating community by analysing the demethylating bacterial community beyond the commonly used subclade level and found a high diversity of closely related oligotypes with distribution patterns that tentatively indicate a wide range of niches. However, we did not see differences of lifestyles (particle attached vs. free living) at the oligotype level, but rather at the coarser subclade level. A broad potential for DMSP demethylation is of particular importance in highly dynamic systems such as coastal areas. The confirmation of the existence of low and high affinity ecotypes, however, requires further experimental approaches. We suggest that seasonality as well as DMSP availability are the main factors shaping the demethylating bacterial community in both MS and the AW in the coastal Mediterranean Sea.

## Supporting information


**Appendix S1:** Supplementary Information

**Figure S1:** Depth‐integrated abundance of phytoplankton groups over the upper 10 m water column.

**Figure S2:** Correlation plots of the most abundant oligotypes of phylogenetic groups representative of *dmd*A harbouring bacteria and DMSPt concentrations. Correlation plots of Rhodospirillales oligotypes (A/2) are shown in brown, SAR11 oligotypes (subclade B/4) in turquois and OM60 oligotypes (subclade E/2) are shown in purple. Correlation coefficient (r) and p values are indicated, correlation plots with p values ≤0.05 are in bold.

**Figure S3:** Box‐plot of leucine incorporation into heterotrophic microbes (median, 10^th^, 25^th^, 75^th^ and 90^th^ percentiles) in marine snow (grey) and ambient water (white).

**Figure S4:** Box‐plot of prokaryotic abundance (median, 10^th^, 25^th^, 75^th^ and 90^th^ percentiles) in marine snow (grey) and ambient water (white).

**Figure S5:** Map of sampling area in the northern Adriatic Sea. The diamond marks the location of station RV001 where MS, AW and phytoplankton samples were collected seasonally.

**Table S1:** Excel file

**Table S2:** Primer pairs used for amplification of *rec*A, 16S rRNA gene and five specific *dmd*A subclades. Annealing temperatures for PCR and qPCR, and efficiency of the qPCR are indicated.
**Table S3:** Characteristics of the NGS dataset. Singletons and reads that appeared in the negative control were subtracted from the presented values. Percent of reads indicates the percentage of reads remaining after quality filtering as compared to the original reads in each sample. The asterisk indicates that the sample was removed from the analysis due to low number or percentage of reads after quality filtering, as indicated in the Methods section.
**Table S4:** Diversity indices of the NGS dataset.

**Video S1:** Supplementary video.
