# Supplementary material for: Seasonal dynamics of marine snow‐associated and free‐living demethylating bacterial communities in the coastal northern Adriatic Sea
Source: Environ Microbiol Rep. 2019 Jul 25;11(5):699–707. doi: 10.1111/1758-2229.12783 (PMC6771949; doi:10.1111/1758-2229.12783)
Supplement: Supplementary file 1 — Appendix S1: Supplementary Information [file EMI4-11-699-s001.docx]

**Experimental procedures**

*Sampling*

Marine snow (MS) and ambient water (AW) were collected about 1 km off the coast of Rovinj, Croatia (45.08347°N, 13.60518°E) (Fig. S5) at 15 ± 2 m depth in June 2015 (8 days), November 2015 (10 days), February 2016 (8 days), May 2016 (7 days) and July 2016 (8 days), thus covering a full seasonal cycle (summer, fall, winter, spring, summer respectively). Each sampling campaign consisted of seven to ten consecutive sampling days (Table S1). MS was collected with 100 mL syringes (0.1M HCl-rinsed prior to use) at 15 ± 2 m depth by SCUBA diving as described previously (Herndl and Peduzzi, 1988; Rath et al., 1998) (supplementary video). Although MS particles were selectively collected, a small amount of AW was collected along with the MS. Therefore in this study the term ‘marine snow’ includes the highly porous MS particle and the water in close proximity to it. Separating MS and AW without loosing the pore-water, which includes biological and chemical compounds of the MS, is difficult as there are no clear biological and chemical boundaries. The bacterial community is not strictly separated into free-living and particle-attached bacteria, but rather into a gradient of particle association and sporadic attach- and detachment of bacteria (Gibiansky et al., 2010; Salazar et al., 2015; Son et al., 2015), which are included in MS as sampled via syringes. Due to its fragile and sticky nature, MS changes in size after collection, making a volume determination and quantification difficult. However, due to the narrow opening of the syringe (~3mm diameter) MS was sampled with high efficiency. For each sampling day, the MS collected in the different syringes was pooled into a pre-rinsed glass bottle and stored in the dark at in situ temperature until further processing (~30 min) in the lab. The pooled MS sample is considered representative for the quantity and quality of the MS at the day of sampling, as exact size and volume determination was not possible. However, various concentrations determined in MS therefore represent conservative values and were compared with that on the AW. All measurements on MS were done on subsamples of the pooled MS. Care was taken to homogenize the MS particles by carefully mixing and slowly inverting the glass bottle containing the pooled MS before collecting subsamples. Due to the heterogeneous nature of MS particles, however, the standard deviations of measurements on MS are generally higher than those of the AW. AW was collected with 5 L Niskin bottles at the same depth as MS and filled into 0.1M HCl-rinsed 2L polycarbonate bottles. The collected AW includes a small proportion of MS particles, however large and fast sinking particles were naturally omitted as they sink into the space below the spout of the niskin bottle (Suter et al., 2017). The samples were transferred to the laboratory of the Center for Marine Research at Rovinj (Ruder Bošković Institute) within 30 min for further processing as detailed below.

*Inorganic nutrient analyses*

Inorganic nutrient analyses of NO_3_^-^, NO_2_^-^, Si, NH_4_^+^, PO_4_^3-^ and DOP were performed only on AW samples. Seawater samples were filtered through pre-combusted Whatman GF/F filters and stored in polyethylene bottles at -20°C. Analyses were performed within one month following standard protocols (Strickland and Parsons, 1972; Ivančič and Degobbis, 1984).

*Phytoplankton abundance and community composition*

To determine phytoplankton community composition 200 mL samples were collected at 0m, 5m and 10m depth (except in November 0 and 5m only) on 15 Jun 2015, 15 Nov 2015, 20 Feb 2016, 06 May 2016 and 19 Jul 2016. Phytoplankton samples were fixed with neutralised formaldehyde (2% final concentration). Phytoplankton were counted in 50 mL subsamples after allowing the cells to settle for 40 h (Hasle, 1978) using an Axiovert 200 inverted microscope (Zeiss GmbH, Oberkochen, Germany) and following the Utermöhl (1958) method. Phytoplankton community composition was determined using standard identification keys in the laboratory of the Center for Marine Research (Ruder Bošković Institute). The measured phytoplankton abundance was integrated over the upper 10m water column using the trapezoidal approach.

*Prokaryotic abundance*

Ten mL of MS and AW were fixed with 0.2 µm-filtered formaldehyde (final concentration 2%) and stored overnight at 4°C. Subsequently, the samples were filtered at low pressure (< 0.2 bar) onto 0.2 µm GTTP polycarbonate filters (Millipore, filter diameter 25 mm) supported by 0.45 µm HAWP (Millipore) filters. The filters were air-dried and stored in 2 mL cryovials (Biozym) at -80°C until analysis in the home lab. The filters were stained with DAPI (4’, 6-diamidino-2-phenylindole, 2 μg mL^-1^ final concentration) for 10 min and observed under an epifluorescence microscope (Axio Imager M2, Carl Zeiss, 1,250x magnification). DAPI-stained cells were counted in 20 randomly selected fields of view per sample with > 200 cell counts per sample. The field of view was 1.72 x 10^3^ µm^2^.

*Leucine incorporation into prokaryotic biomass*

Leucine incorporation as a proxy for heterotrophic biomass production of prokaryotes was measured using 1.5 mL of MS or AW sample following the protocol of Smith and Azam (1992). Briefly, four replicates and two trichloroacetic acid (TCA)-killed blanks were amended with 40 nmol L^-1^ (final concentration) of [^3^H]-leucine (specific activity 120 Ci mmol^-1^, BioTrend Chemicals) and incubated at in situ temperature in the dark for 2-4 h. Incubations were terminated by adding TCA (5% final concentration). Subsequently, the samples were centrifuged at 20,000x *g* for 10 min. The supernatant was discarded, while the resulting pellet was rinsed with TCA (5%), centrifuged (20,000x *g*, 10 min) and 1mL of Ultima-GOLD (Canberra-Packard) scintillation cocktail added to the pellet. After 18 h, the samples were measured in a liquid scintillation counter (Canberra Packard TriCarb 2900 TR, Perkin Elmer Packard, USA). The samples were corrected for quenching and the mean of the disintegrations per minute (DPM) of the TCA-killed blank was subtracted from the mean of the live samples. The resulting DPM were converted into leucine incorporation rates.

*DMSP*

MS and AW were carefully filled into 10 mL crimp-top glass vials leaving no headspace. Duplicate samples for total DMSPt + DMS measurements (particulate DMSP + dissolved DMSP + DMS) were fixed by adding one NaOH pellet. Samples were stored at room temperature in the dark until analysis at the Institute of Marine Sciences (CSIC, Barcelona, Spain). DMSPt + DMS concentrations were measured using a purge-and-trap method and sulfur-specific gas chromatography as described elsewhere (Galí et al., 2011).

*DNA extraction*

One liter of AW and 150 to 500 mL of MS were filtered onto 0.2 µm polyethersulfone filters (47 mm diameter, Supor, PALL Gelman) using an aspirator pump (Cole-Parmer) immediately after sampling. The filters were stored in cryovials (Biozym), flash-frozen in liquid nitrogen and subsequently stored at -80°C until DNA extraction in the home lab. DNA was extracted using the standard phenol-chloroform method with slight modifications as described below.

Briefly, the filters were thawed and cut with sterile scissors into small pieces. After adding 2 mL of lysis buffer (0.2 mL Tris 100 mM pH8, 0.2 mL EDTA 250 mM pH8, 0.04 mL NaCl 5M, 1.56 mL RNAse /DNAse-free water from Sigma-Aldrich) and 8.75 U/mL final concentration of Lysozyme Ready-Lyse (Epicenter), the filters were incubated at 37°C for 45 min. Subsequently, sodium dodecyl sulfate (1% final concentration) and 8 U/mL of proteinase K (from *Tritirachium album*, Sigma-Aldrich) were added and the samples incubated at 55°C for 1 h. Combusted (450°C) zirconium beads (0.1 mm diameter Zirconia/Silica, BioSpec Products) were added to the samples and the tubes were vortexed at maximum speed for 10 min. Samples were heated up to 70°C for 30 min and subsequently, the lysate was aspirated and split into two 2 mL microcentrifuge tubes. An equal volume of water-saturated phenol pH 8 (Sigma-Aldrich) was added to the samples, mixed and centrifuged for 10 min. All centrifugation steps were done at 21,000x *g* at 4°C. The aqueous layer was recovered and an equal volume of PCI (phenol-chloroform-isoamylalcohol 25:24:1, Sigma-Aldrich) was added, mixed and centrifuged for 10 min. The aqueous layer was aspirated, mixed with an equal amount of ice-cold chloroform (Sigma-Aldrich) and centrifuged for 10 min. Subsequently, DNA from the aqueous layer was precipitated by adding 10 µL of 5 M NaCl and 2 volumes of 100% ethanol (Merk) at -20°C overnight, followed by centrifugation for 25 min. The pellet was washed with 70% ethanol and centrifuged for 20 min. Thereafter, the pellet was dried in a Concentrator plus (Eppendorf) at 45°C for 30 min. The split pellets from each sample were re-suspended in 50 µL of RNAse/DNAse-free water, pooled and stored at -80°C until analysis.

*Preparation of qPCR standards*

Analysis of the demethylating bacterial community was done using the primer sets described by Varaljay et al. (2010) (Table S2) targeting different clades (A to E) of the *dmd*A gene-harboring bacteria. Clade A is represented by *Roseobacter* and Rhodospirillales species, clade B by the SAR116 group member “*Candidatus* Puniceispirillum marinum”. Clade C is represented by the SAR11 strain *Pelagibacter ubique* HTCC7211, and clade D by the SAR11 strains *Pelagibacter ubique* HTCC1002, HTCC1062 and another homolog to HTCC7211. Clade E includes sequences from the marine Gammaproteobacterium clade OM60/NOR5 clade (thereafter referred to as OM60 clade). *Dmd*A genes of all described subclades were PCR amplified in a Master cycler (Eppendorf) via gradient PCR with the following settings: initial denaturation at 94°C for 2 min, followed by 35 cycles of 94°C for 20 s, annealing at a gradient from 40°C to 60°C for 30 s, extension at 68°C for 30 s, followed by a final extension step at 68°C for 10 min and cooling at 4°C. Only those subclades with positive PCR were further used for qPCR analysis. PCR of the *rec*A gene was carried out with the following settings: 4 min initial denaturation at 94°C, 30 cycles at 94°C for 30 s, annealing at 53°C for 30 s, extension at 72°C for 45 s, followed by a final extension at 72°C for 10 min and cooling at 4°C. Each 25 µL reaction of the Master-mix contained 1.25 U/µL picomaxx high fidelity polymerase and 2.5 µL of 10 x picomaxx buffer (Agilent Technologies), 2.5 µL dNTPs (dNTP mix containing 2 mM of each dNTP resulting in 0.2 mM final concentration), 0.5 µL of 20 mg mL^-1^ BSA, 0.5 µL of 25 mM MgCl_2_ (Thermo Scientific) and 0.5 µM of each primer. The PCR products were purified with the PCRExtract MiniKit (5-PRIME) and the DNA concentration was measured with a Nanodrop® spectrophotometer. Standards for the *dmd*A genes A/2-sp, B/3-sp, B/4-sp, D/3-sp and E/2-sp and the *rec*A gene (Holmes et al., 2004) (Table S2) were prepared from the purified PCR products. The gene abundance was calculated from the concentration of the purified DNA and the size of the fragment. Serial dilutions were prepared for each gene with TRIS buffer (10 mM, pH 8.0) from 10^7^ to 10^0^ gene copies per mL and added as a standard in triplicate to each qPCR run.

*qPCR analysis*

The samples were loaded in triplicate and duplicate (*rec*A and *dmd*A, respectively) to a 96-well qPCR plate (Bio-Rad), closed with optical tape (Bio-Rad) and run on a Light Cycler 480 (Roche). The AW samples were diluted 1:10 and the MS samples to 1:100 with RNAse/DNAse-free water. The reaction mixture for each sample contained 1x Mastermix (LightCycler 480 SYBR Green I Master, Roche), 0.5 µM of each primer, 1 µL of diluted sample and ultrapure sterile water (Roche) made up to 10 µL. Thermocycling for *rec*A and *dmd*A genes was initiated by a denaturation step at 95°C for 10 min and followed by 50 cycles consisting of a denaturation step at 95°C for 5 s; annealing at specific temperatures for each gene and subclades (Table S2) for 5 s and for 10 s for *rec*A and *dmd*A subclades*,* respectively, extension at 72°C for 15 s, and a plate read at 74°C for 3 s.

Quantitative PCR analysis was performed on all samples for *rec*A and on at least three samples from each sampling campaign for the *dmd*A subclades. The total *dmd*A gene abundance was calculated as the sum of all *dmd*A gene subclades. The sub-clade specificity of the primers was assessed *in silico* and experimentally by Varaljay et al. (2010). The total *dmd*A gene abundance and the abundance of each subclade are presented as their respective ratio to *rec*A gene abundance. Normalization to *rec*A was used as a proxy of bacterial abundance and to account for possible variations in DNA extraction efficiency between different samples. The housekeeping gene *rec*A is commonly used for this purpose in qPCR studies, as it is a single copy gene present in all bacteria (Miller and Kokjohn, 1990).

*Bacterial community composition assessed by next generation sequencing*

The 16S rRNA genes of the bacterial community inhabiting MS and the AW were PCR-amplified from samples collected at the beginning, middle and end of each sampling period using a Mastercycler (Eppendorf) (in total 30 samples). One negative control (RNAse/DNAse-free water) was also PCR amplified. Thermocycling consisted of a 1^st^-step PCR with the primers 341_ill forward and 802_ill reverse containing adaptors (Table S2) and a KAPAHiFi Mastermix (Peqlab) with the following program: initial denaturation at 94°C for 3 min, followed by 20 cycles of 94°C for 30 s, annealing at 56°C for 30 s, extension at 72°C for 90 s; a final extension step was done at 72°C for 7 min followed by cooling at 4°C. PCR products were purified with Agencourt AMPure XP magnetic particles (Beckman Coulter) and quantified with a Quant-IT PicoGreen® Assay (Invitrogen). The Nextera 2^nd^-step PCR was performed under the same thermocycling conditions as described above for 10 additional cycles, followed by pooling and 2 x 250 v2 sequencing on an Illumina MiSeq system (Microsynth AG, Balgach, Switzerland).

*Sequence data processing and analysis*

The 16S rRNA sequences were analyzed using the program Mothur (Schloss et al., 2009) against the Silva v128 reference database with a cut-off value of 80. Operational taxonomic units (OTUs) were defined as sequences with 97% similarity. OTUs sequences identified in the negative control were subtracted from the OTUs. Also, singletons were removed. Samples with less than 1000 reads or with less than 80% of the reads remaining in each sample after quality control were removed from the dataset. This procedure resulted in the exclusion of 9 out of the 30 samples from further analysis (Table S3). The community composition is given as percentage of the total community in each sample to allow comparison between different samples.

Oligotyping (Eren et al., 2013) was performed following the “best practices” pipeline of A. Murat Eren (http://merenlab.org/2013/11/04/oligotyping-best-practices/). The reads of interest were extracted using the “mothur2oligo” script (courtesy of Michelle Berry) after running the Mothur pipeline (MiSeq SOP) until the taxonomy assignment step, and skipping the “pre-clustering” step to avoid the removal of unique sequences. Oligotyping was carried out at the taxonomic level of the order. Shannon entropy level of 0.2 was used to recover true and relevant ecological oligotypes and omit random sequencing errors. The minimum substantive abundance of an oligotype (-M parameter) was set to 50. This method revealed a large set of oligotypes for each OTU. However, further analysis was done only on those oligotypes with an overall read abundance of more than 1000. We focused on the representative bacterial groups of those *dmd*A subclades that appeared throughout the seasonal cycle or in both sample types (MS and AW). We linked the relative abundances of the *dmd*A subclades determined by qPCR to the relative abundances of oligotypes of the bacterial community and to DMSPt + DMS concentrations. This approach was used to provide insights into fine scale differences of highly similar bacterial groups and their ecological relevance as ecotypes.

*Statistical analysis*

All statistical analyses were conducted with the software Past 3.15 (Hammer et al., 2001) and SigmaPlot Version 13. A t-test was used if a normal distribution was verified. Otherwise, a Kruskal-Wallis test was used to compare MS versus AW samples and Mann-Whitney pairwise post-hoc test to compare sampling months within MS or AW samples. Differences were considered statistically significant if *p* < 0.05. All values are expressed as mean ± SD if not stated otherwise. The enrichment factor (EF) is presented as monthly average ratio of the respective value in MS to that in the AW. A canonical correspondence analysis (CCA) was used to analyze the temporal distribution patterns of the bacterial community in response to environmental parameters. The integrated abundance of phytoplankton groups, DMSPt + DMS concentrations, *dmd*A subclades and oligotypes were used as environmental variables. The *dmd*A subclades and the different oligotype abundances are expressed as percentage of their respective total. The MS samples exhibited an extremely high relative abundance of *Synechococcus* in February (70%). Due to this high abundance and its un-relatedness to demethylating bacteria, *Synechococcus* was excluded from the CCA analysis in the MS and AW dataset.

Supplementary results

*Environmental parameters*

Marine snow (MS) was present in all sampling months ranging from 0.5 to 5 mm in diameter as estimated by *in situ* visual examination. On average, 71 ± 9 MS particles were collected in each of the 100-mL disposable syringes.

The averaged abundance of prokaryotes was higher in MS (8.45 x 10^5^ ± 3.29 x 10^5^ cells mL^-1^) than in AW (5.42 x 10^5^ ± 2.87 x 10^5^ cells mL^-1^) with enrichment factors ranging from 1 to 4 (Fig. S5, Table S1). Leucine incorporation in MS showed the same trend as in the AW, however MS (7.75 ± 13.25 nmol Leu L^-1^ h^-1^ averaged over all samples) exhibited significantly higher leucine incorporation than AW (2.04 ± 1.02 nmol Leu L^-1^ h^-1^ averaged over all samples). The EF of leucine incorporation ranged from 1 to 7 (Fig. S3, Table S1). Cell-specific leucine incorporation was higher in MS than in AW in June (28.65 ± 6.54 amol Leu cell^-1^ h^-1^, EF=5) and July (19.63 ± 18.30 amol Leu cell^-1^ h^-1^, EF=2). In contrast, from November to May cell-specific leucine incorporation was similar in MS and AW (data not shown).

*rec*A *gene abundance and dmd*A *subclades determined by qPCR*

The *rec*A gene abundance in MS was not significantly different from that in AW. The EF ranged from 0.2 in February to 3 in June. Bacterial abundance as determined by *rec*A gene abundance in MS and AW was higher in June than in all other months (Table S1).

*Bacterial community composition*

The number of reads after quality control and removal of singletons ranged from 3,056 to 64,244 per sample, and the sequencing coverage ranged from 0.98 to 1 (Table S3). Bacterial community diversity indices were similar for MS and AW. The highest diversity (Simpson and Shannon diversity index) for both AW and MS was found in November and the lowest in February (Table S4). Of the 20 most abundant OTUs in the AW and MS, *Synechococcus* and Flavobacteria mainly of the genus marine group NS4 dominated the bacterial community (Fig. 3A, B). Besides *Synechococcus* and Flavobacteria, the representative bacterial groups of the *dmd*A subclades were highly abundant in the AW, particularly the SAR11 clade and Rhodospirillales. The SAR11 clade was particularly abundant in the AW in November and February with *Candidatus* Pelagibacter being the most abundant OTU. Bacteria of the order Rhodospirillales were most abundant in June. The SAR116 clade was most abundant in June, May and July in the AW (Fig. 3A). Within the 20 most abundant OTUs in MS, Vibrionales and Planctomycetes were highly abundant. The SAR11 clade was also abundant in MS particularly in November (Fig. 3B).
