# Supplementary figures and images for: Seasonal dynamics of marine snow‐associated and free‐living demethylating bacterial communities in the coastal northern Adriatic Sea
Source: Environ Microbiol Rep. 2019 Jul 25;11(5):699–707. doi: 10.1111/1758-2229.12783 (PMC6771949; doi:10.1111/1758-2229.12783)

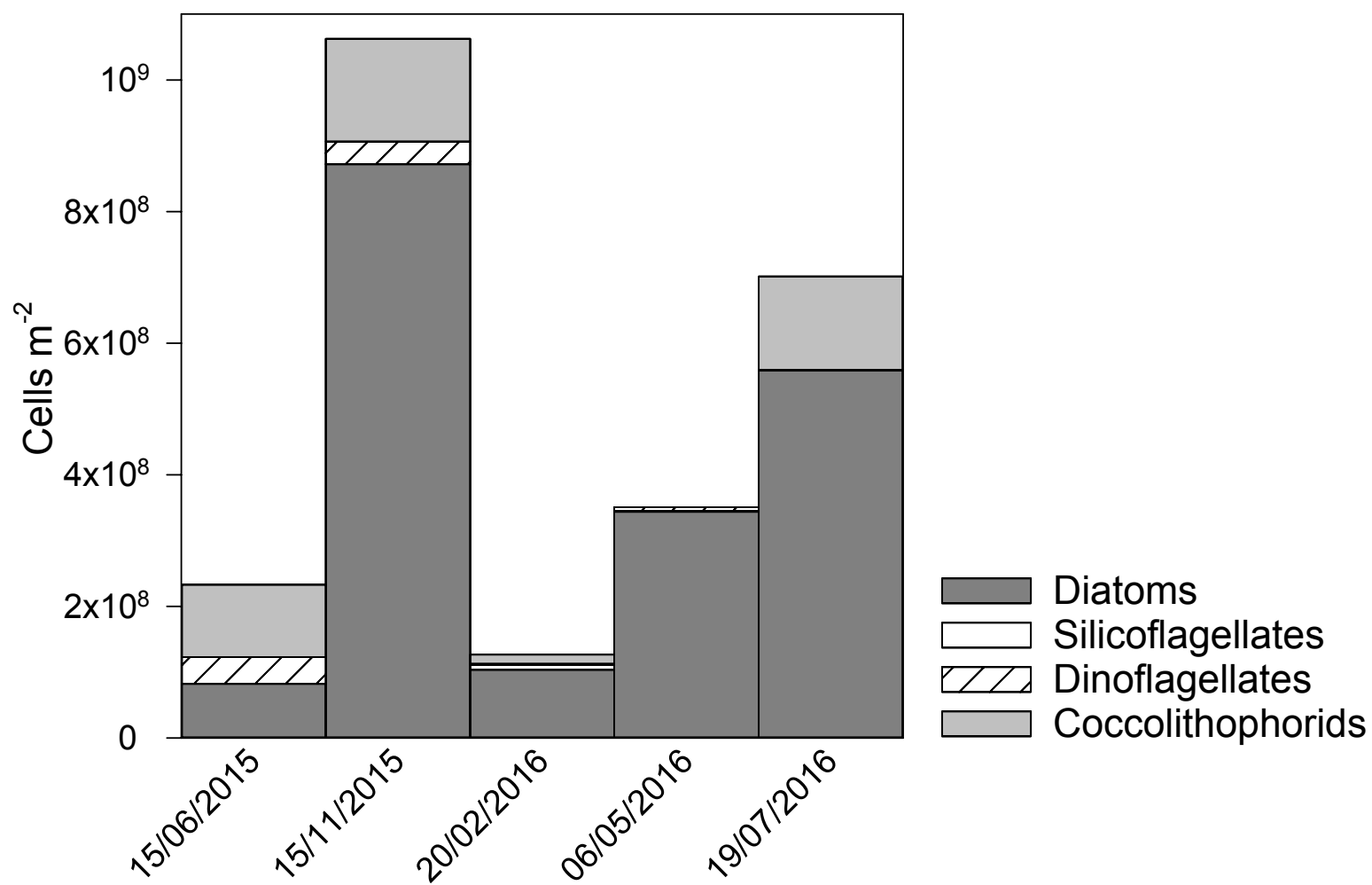

Supplement: Supplementary file 2 — Figure S1: Depth‐integrated abundance of phytoplankton groups over the upper 10 m water column. [file EMI4-11-699-s002.PDF]

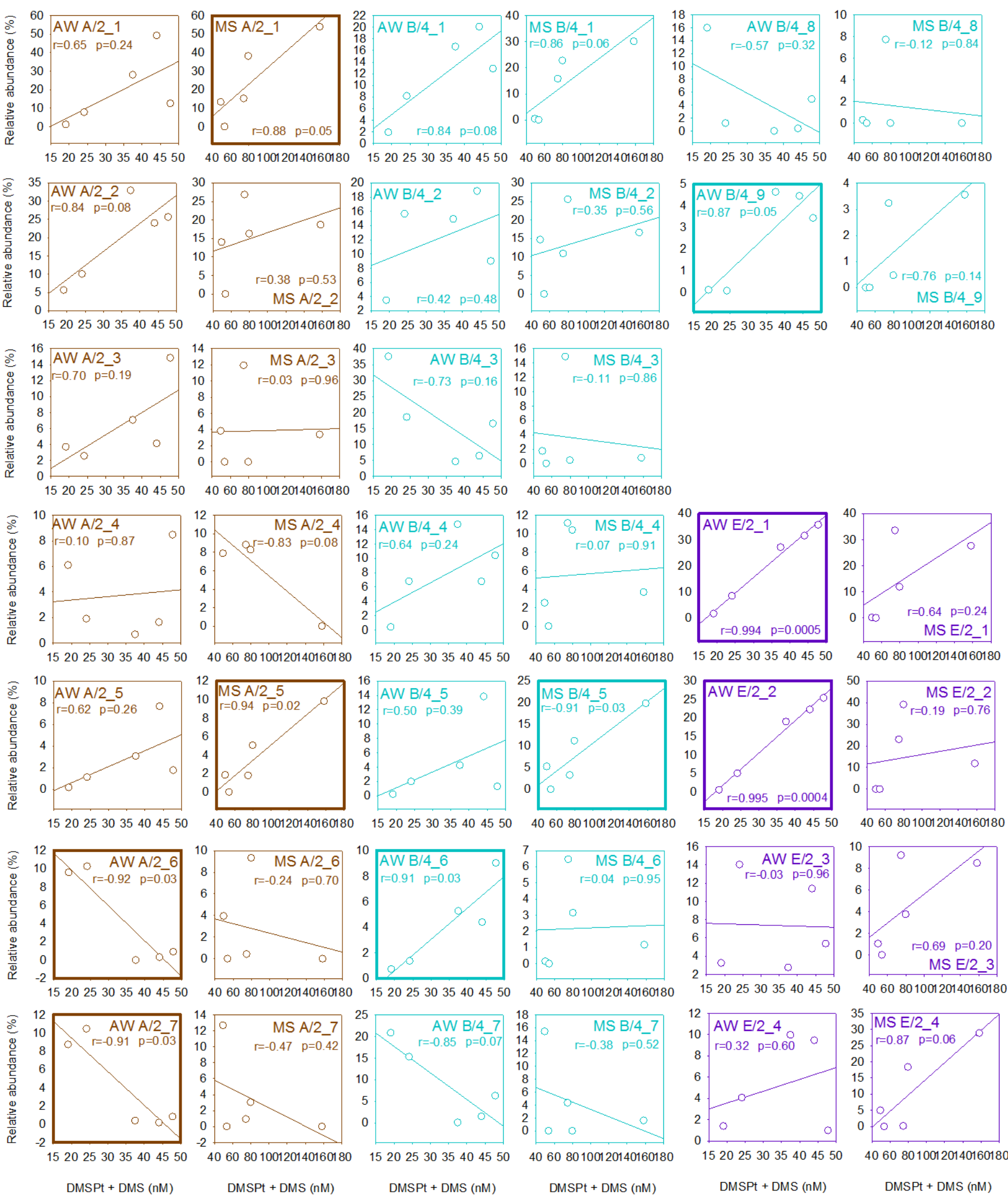

Supplement: Supplementary file 3 — Figure S2: Correlation plots of the most abundant oligotypes of phylogenetic groups representative of dmdA harbouring bacteria and DMSPt concentrations. Correlation plots of Rhodospirillales oligotypes (A/2) are shown in brown, SAR11 oligotypes (subclade B/4) in turquois and OM60 oligotypes (subclade E/2) are shown in purple. Correlation coefficient (r) and p values are indicated, correlation plots with p values ≤0.05 are in bold. [file EMI4-11-699-s003.PDF]

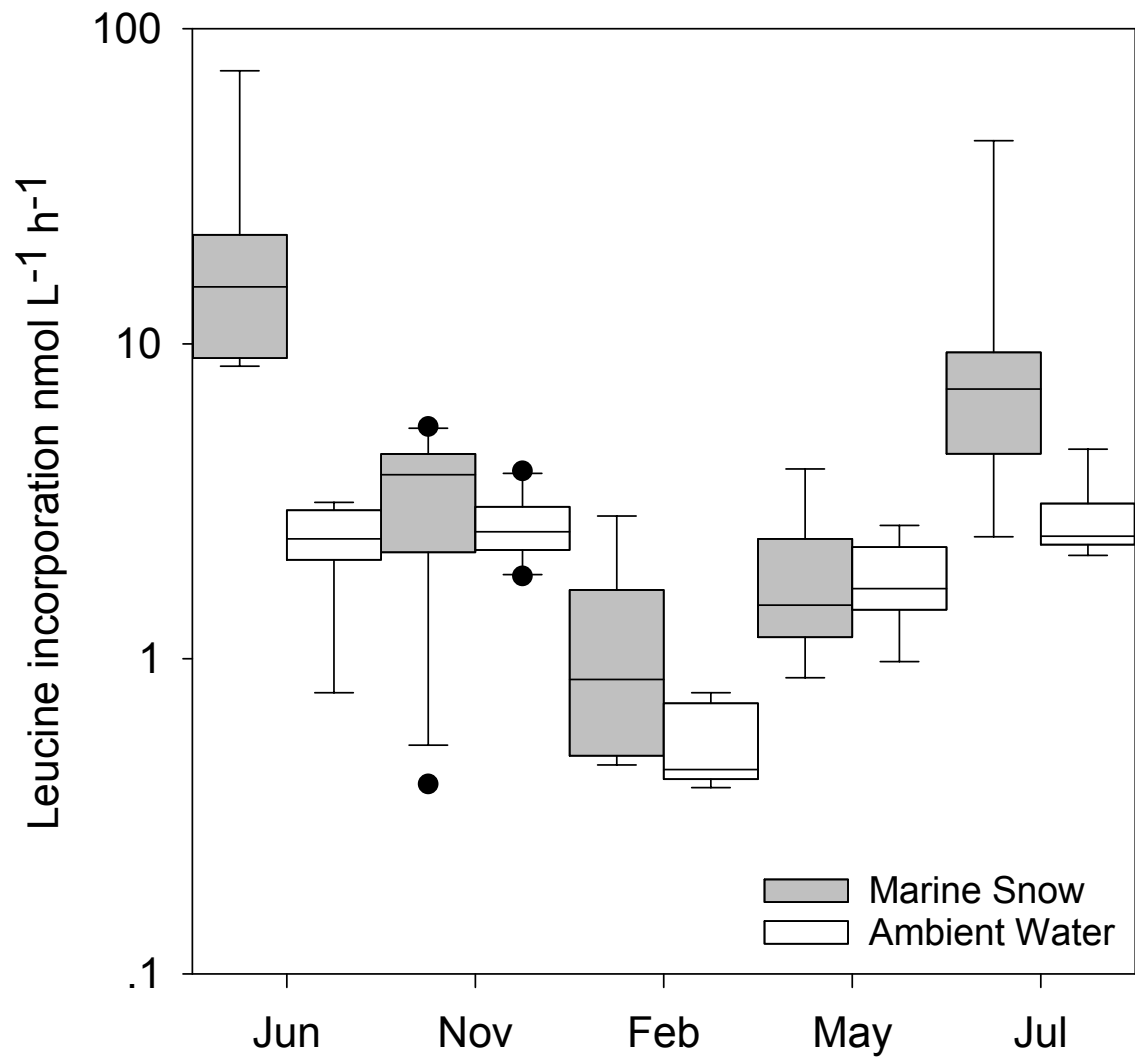

Supplement: Supplementary file 4 — Figure S3: Box‐plot of leucine incorporation into heterotrophic microbes (median, 10th, 25th, 75th and 90th percentiles) in marine snow (grey) and ambient water (white). [file EMI4-11-699-s004.PDF]

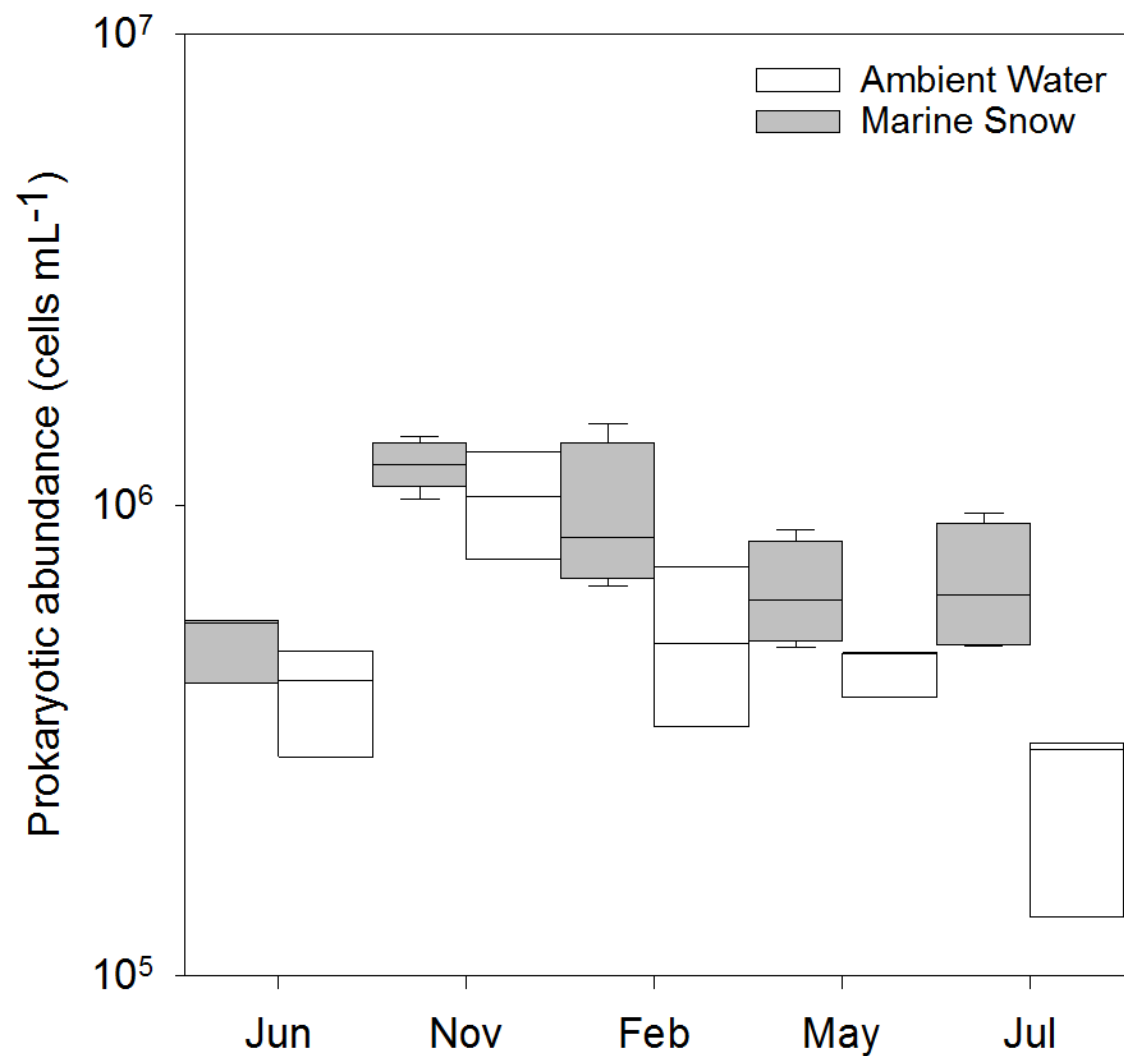

Supplement: Supplementary file 5 — Figure S4: Box‐plot of prokaryotic abundance (median, 10th, 25th, 75th and 90th percentiles) in marine snow (grey) and ambient water (white). [file EMI4-11-699-s005.PDF]

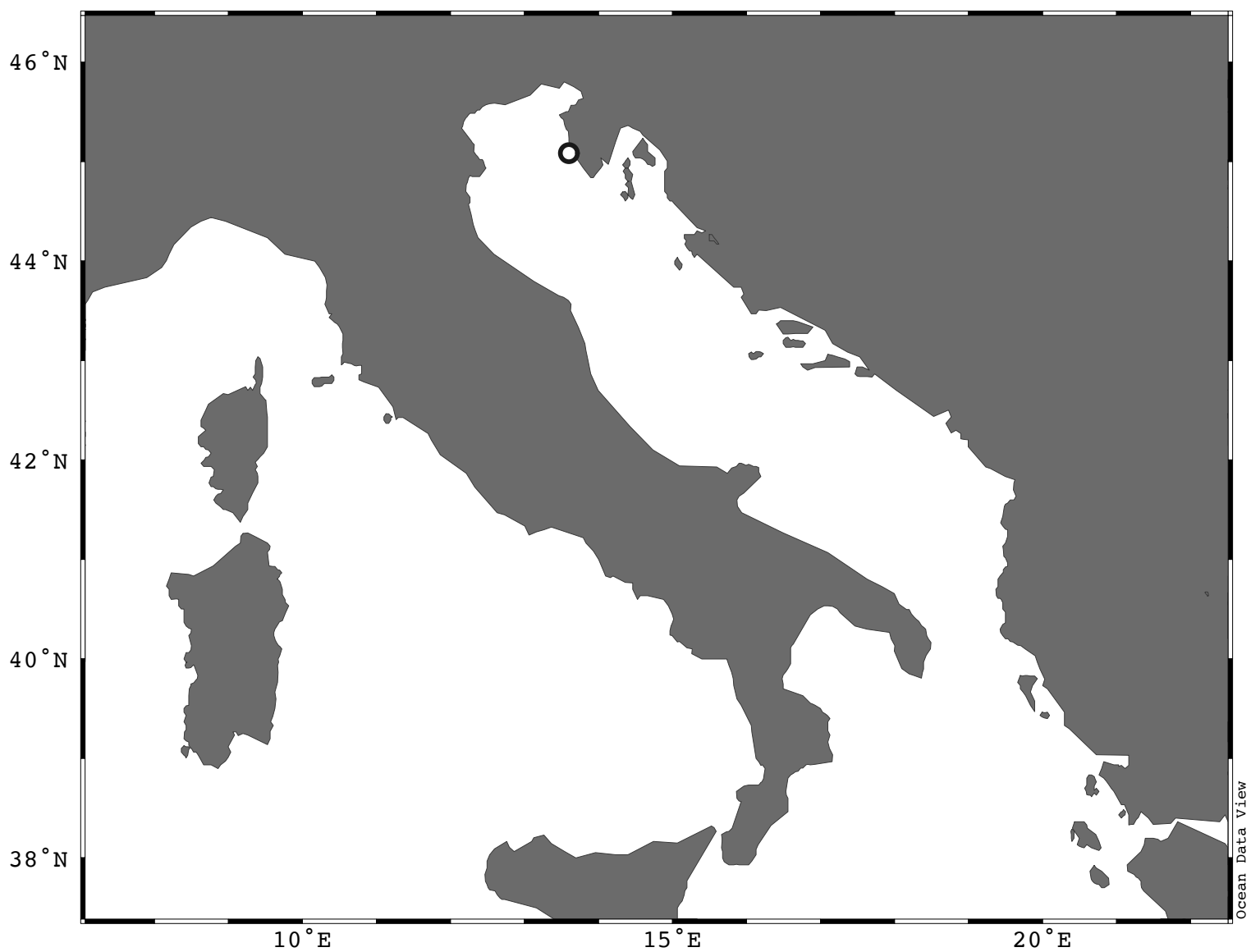

Supplement: Supplementary file 6 — Figure S5: Map of sampling area in the northern Adriatic Sea. The diamond marks the location of station RV001 where MS, AW and phytoplankton samples were collected seasonally. [file EMI4-11-699-s006.PDF]
