# Supplementary material for: Seasonal dynamics of marine snow‐associated and free‐living demethylating bacterial communities in the coastal northern Adriatic Sea
Source: Environ Microbiol Rep. 2019 Jul 25;11(5):699–707. doi: 10.1111/1758-2229.12783 (PMC6771949; doi:10.1111/1758-2229.12783)
Supplement: Supplementary file 8 — Table S2: Primer pairs used for amplification of recA, 16S rRNA gene and five specific dmdA subclades. Annealing temperatures for PCR and qPCR, and efficiency of the qPCR are indicated. Table S3: Characteristics of the NGS dataset. Singletons and reads that appeared in the negative control were subtracted from the presented values. Percent of reads indicates the percentage of reads remaining after quality filtering as compared to the original reads in each sample. The asterisk indicates that the sample was removed from the analysis due to low number or percentage of reads after quality filtering, as indicated in the Methods section. Table S4: Diversity indices of the NGS dataset. [file EMI4-11-699-s008.pdf]

Table S2. Primer pairs used for amplification of *rec*A, 16S rRNA gene and five specific *dmd*A subclades. Annealing temperatures for PCR and qPCR, and efficiency of the qPCR are indicated.

Gene/subclade	Position	Amplicon length (bp)	Primer sequence 5' to 3'	Annealing temp (°C) PCR	Annealing temp (°C) qPCR	Efficiency (%)	Reference
*rec*A		212	RECAF: TGTGCITTTATWGATGCIGAGCATGC<br>RECAR: CCCATGTCICCTTCKATTTCIGCTTT	53	53	91 - 93.5	Holmes et al. 2004
A/2-sp	339 - 486	147	A/2-spFP: CGATGAACATTGGTGGGTTTCTA<br>A/2-spRP: GCCATTAGGTCGTCTGATTTTGG	59 - 62	62	93.5	Varaljay et al. 2010
B/3-sp	169 - 323	154	B/3-spFP: GATGTCTCCTGCCAACGTCAGGTCGA<br>B/3-spRP: ACCGGGTCATTGATCATGCCTGCG	62	62	88.5	Varaljay et al. 2010
B/4-sp	361 - 553	192	B/4-spFP: ATTGCCGACTCGGATGTTCT<br>B/4-spRP: CAAGAAGGTCAAACATGGCAAAC	58 - 62	62	93	Varaljay et al. 2010
D/3-sp	347 - 473	126	D/3-spFP: AATGGTGGATTTCTATTGCAGATAC<br>D/3-spRP: GATTTTGGACCTTGTACAGCCA	54	54	97.5	Varaljay et al. 2010
E/2-sp	154 - 287	133	E/2-spFP: CATGTTCAGATCTGGGACGT<br>E/2-spRP: AGCGGCACATACATGCACT	57 - 62	62	94	Varaljay et al. 2010
16S rDNA	V34	460	341ill NGS forward: TCGTCGGCAGATGTGTATAAGAGACAGCCTACGGGNGGCWGCAG<br>802 ill NGS reverse: GTCTCGTGGGCTCGGAGATGTGTATAAGAGACAGGACTACHVGGGTATCTAATCC	56			Illumina

For Peer Review Only

Table S3. Characteristics of the NGS dataset. Singletons and reads that appeared in the negative control were subtracted from the presented values. Percent of reads indicates the percentage of reads remaining after quality filtering as compared to the original reads in each sample. The asterisk indicates that the sample was removed from the analysis due to low number or percentage of reads after quality filtering, as indicated in the Methods section.

Sample	Coverage	Nr of reads	Percent of reads
AW_24/6/2015	1.00	42892	98.7
AW_25/6/2015	1.00	43523	99.0
AW_26/6/2015	1.00	29405	98.5
AW_3/11/2015	1.00	43517	98.9
AW_7/11/2015*	0.99	1874	71.1
AW_11/11/2015*	0.83	78	44.1
AW_1/2/2016	1.00	50794	99.1
AW_3/2/2016	1.00	45193	98.9
AW_8/2/2016	1.00	52662	99.0
AW_9/5/2016	0.98	3056	90.6
AW_12/5/2016	1.00	32792	98.7
AW_16/5/2016	1.00	64244	99.1
AW_27/07/2016*	0.97	371	19.4
AW_28/07/2016*	0.98	509	30.7
AW_30/7/2016	1.00	17873	92.5
MS_24/6/2015	1.00	41855	94.8
MS_25/6/2015	1.00	41875	96.0
MS_26/6/2015	1.00	22969	92.8
MS_3/11/2015	1.00	21624	91.0
MS_7/11/2015	1.00	8734	84.3
MS_11/11/2015*	0.86	160	55.6
MS_1/2/2016	1.00	8235	84.8
MS_3/2/2016	1.00	9303	85.8
MS_8/2/2016*	1.00	5012	69.1
MS_9/5/2016*	1.00	1543	46.4
MS_12/5/2016	1.00	33053	95.1
MS_16/5/2016	1.00	39617	95.4
MS_27/07/2016*	0.99	648	38.5
MS_28/07/2016*	0.99	849	32.3
MS_30/7/2016	1.00	42422	94.2

For Peer Review Only

Table S4. Diversity indices of the NGS dataset.

Sample	Simpson index	Shannon index	Evenness
AW_24/6/2015	0.9	3.4	0.1
AW_25/6/2015	0.9	3.2	0.1
AW_26/6/2015	0.9	3.4	0.1
AW_3/11/2015	1.0	4.0	0.2
AW_7/11/2015	-	-	-
AW_11/11/2015	-	-	-
AW_1/2/2016	0.9	3.1	0.1
AW_3/2/2016	0.9	3.1	0.1
AW_8/2/2016	0.9	3.8	0.1
AW_9/5/2016	-	-	-
AW_12/5/2016	0.9	3.1	0.1
AW_16/5/2016	0.9	3.3	0.1
AW_27/07/2016	-	-	-
AW_28/07/2016	-	-	-
AW_30/7/2016	0.9	3.6	0.2
MS_24/6/2015	0.9	3.3	0.2
MS_25/6/2015	0.9	3.8	0.2
MS_26/6/2015	0.9	3.3	0.2
MS_3/11/2015	1.0	4.0	0.4
MS_7/11/2015	1.0	4.2	0.5
MS_11/11/2015	-	-	-
MS_1/2/2016	0.6	1.8	0.1
MS_3/2/2016	0.5	1.6	0.1
MS_8/2/2016	0.8	2.6	0.3
MS_9/5/2016	-	-	-
MS_12/5/2016	0.9	3.7	0.2
MS_16/5/2016	1.0	3.7	0.2
MS_27/07/2016	-	-	-
MS_28/07/2016	-	-	-
MS_30/7/2016	0.9	3.6	0.3

For Peer Review Only
